# Newly qualified professional nurses’ experiences providing postoperative care to children in CTICU

**DOI:** 10.4102/curationis.v47i1.2493

**Published:** 2024-03-29

**Authors:** Thereza M. Mehlape, Sidwell Matlala

**Affiliations:** 1 Department of Nursing, Faculty of Health Sciences, University of Johannesburg, Johannesburg, South Africa

**Keywords:** cardiothoracic intensive care, children, experiences, nursing, post-cardiac surgery, newly qualified professional nurse

## Abstract

**Background:**

It is challenging for newly qualified professional nurses (NQPNs) to care for children with congenital heart abnormalities following cardiac surgery in cardiothoracic critical care units. This population of nurses is allocated to critically ill children in the cardiothoracic intensive care unit (CTICU) even though they lack sufficient knowledge, experience and skills to care for these patients.

**Objectives:**

This study explored, described and made recommendations to support NQPNs who provide postoperative care to children in a CTICU.

**Method:**

A qualitative, exploratory, descriptive and contextual research design was used. Purposive sampling was employed, and in-depth individual phenomenological interviews were conducted with 10 NQPNs. Data were analysed according to Giorgio’s descriptive phenomenological method, and measures to ensure trustworthiness and ethical principles were followed.

**Results:**

The NQPNs cited their lack of knowledge and mentoring as the primary contributors to their perceived challenges. This population requires motivation, mentoring and empowerment to address this phenomenon.

**Conclusion:**

Professional nurses in CTICUs require a supportive work environment, with encouragement from colleagues, management and doctors. A lack of support compromises patient care outcomes and safety, resulting in litigation.

**Contribution:**

Recommendations are provided for nursing education, research and practice to empower NQPNs with knowledge and skills to work with children following cardiac surgery to avoid adverse events in the CTICU.

## Background

Nursing children who have undergone cardiac surgery is challenging for newly qualified professional nurses (NQPNs) in the cardiothoracic intensive care unit (CTICU). The children undergo different types of surgeries to correct cardiac anomalies, and extensive knowledge, and skills are required in operating the various equipment when caring for these critically ill children (Malelo-Ndou, Ramathuba & Netshisaulu 2019:6). The authors further stated that intensive care unit (ICU) is a speciality area that requires competencies in knowledge, skills and values, as well as further training to provide quality care to patients with life-threatening conditions. This view aligns with the South African Nursing Council’s (SANC 2014) emphasis on critical care competencies because critical care units are complex environments that require keen attention to detail during emergencies to save lives.

The CTICU under study experiences a shortage of qualified intensive care nurses, and the healthcare facility consequently allocates lower categories of nurses to the CTICU, compromising quality patient care (Albanesi et al. 2022:205). Furthermore, the authors highlighted that postoperative paediatric cardiac patients in the CTICU need highly skilled professionals to care for them to reduce prolonged stays in the hospital and decrease mortality rates. Nakweenda, Anthonie and Van der Heever (2022:205) reiterated that a shortage of qualified intensive care nurses significantly impacts the provision of quality patient care, which may lead to adverse events in CTICU.

The terms ICU and critical care unit are used interchangeably in this article. In the public academic hospital under study, some NQPNs allocated to critical care units were enrolled under SANC Regulation r. 683, leading to a qualification as a registered nurse. However, in their training to become professional nurses, the r. 683 curriculum (South African Nursing Council 2005) does not cover the complex nursing knowledge and skills required to nurse critically ill children postoperatively in CTICUs.

Ohnstad and Solberg (2017:573, 574) affirmed that Norway faces similar challenges related to a lack of trained professional nurses working in ICUs. The authors further stated that professional nurses need to be ICU trained or experienced in order to care for postoperative children as working in the ICU without expertise is detrimental to patients’ health outcomes and safety. Ohnstad and Solberg (2017:573, 574) concurred that hospitals are burdened by a lack of trained professional nurses, especially nurses trained in the paediatric speciality. Critically ill children’s conditions often change suddenly and require an experienced critical care nurse who can function in a complex and fast-paced, technology-driven ICU environment. Therefore, NQPNs require mentoring and a buddy system to ensure appropriate decision-making and clinical judgement. Hörberg et al. (2018:236) indicated that NQPNs lack the requisite theoretical knowledge (despite their professional knowledge, skills and experience) in critical care emergencies, which are essential to prevent litigations.

## Objective

The purpose of this article is to describe NQPNs’ experiences caring for postoperative paediatric patients in the CTICU of an academic public hospital in Gauteng.

## Methods

A qualitative, exploratory, descriptive and contextual design was used. The 20-bed facility under study admits newborns to 12-year-old children. The majority of patients have cardiac anomalies and receive postoperative care in the CTICU. The target population for the study was 30 professionals meeting specific criteria. Data were collected from one group even though ICU has three categories of professional nurses with different attributes. The group divisions were as follows: 10 professional nurses who had undergone training under SANC Regulation r. 683 (bridging course), working in the CTICU of an academic hospital in Gauteng for less than 1 year with no additional qualifications in critical care nursing. Ten other professional nurses were ICU trained with additional qualifications in Critical Care Nursing Sciences and had worked in the unit for more than 7 years. The unit also consisted of experienced professional nurses with no additional qualifications, but more than 5 years’ experience nursing children in the CTICU. Purposive sampling was used to select participants registered under the SANC Regulation r. 683 (South African Nursing Council 2005) (bridging course) and working in the CTICU of a public academic hospital in Gauteng for less than 1 year with no additional qualifications in critical care nursing.

In-depth phenomenological interviews were conducted (from January 2018 to May 2018) with professional nurses who consented and willingly agreed to participate in the study. The interviews were audio-recorded and lasted approximately 45 min – 60 min. The researcher took field notes to enrich the data (LoBiondo-Wood & Haber 2018:576–577). The interviews were conducted in an office provided by the unit manager; the office was quiet, interviews were conducted at a convenient time for the participants and the researcher ensured their privacy and confidentiality. Ten participants registered under the SANC Regulation r. 683 (bridging course) and working for less than a year in the CTICU of the public academic hospital in Gauteng consented to participate in the study. The central questions posed to participants were as follows: ‘How is it for you nursing children post-cardiac surgery?’ and ‘What can be done to support you?’ Subsequent questions emerged from further probing, like ‘Tell me more about your fears when nursing critically ill children in CTICU’. Data saturation was achieved at the tenth participant when repetitive themes emerged.

### Data analysis

Data were analysed using Giorgi’s thematic data analysis approach. In step one, the researcher listened to the interview recordings and read the transcripts several times to become familiar with the data and get a sense of the whole. The researcher assumed a neutral position during the interviews by applying bracketing. The researcher identified meaning units during step two and regrouped meaning units into clusters in step three. This step was crucial for the researcher to gain a fuller understanding of NQPNs’ lived experiences providing postoperative care to children in a CTICU. The researcher selected elements that were found to be relevant, regrouped and intertwined and established categories. Step four was a continuous process from the previous step, where similar information was grouped together. Lastly, step five entailed the synthetisation and integration of transformed meaning units into statements with themes and subthemes regarding NQPNs’ experiences providing postoperative care to children in a CTICU. After data were analysed, the researcher and the independent coder had a consensus meeting to discuss and agree on the identified themes and sub-themes.

### Trustworthiness

The researcher adopted the following criteria of trustworthiness (Korstjens & Moser 2018:121): credibility, transferability, dependability and confirmability. The study’s credibility was attained through prolonged engagement and triangulation. During data collection, the researcher developed a trusting relationship with the NQPNs by spending sufficient time with them to understand their experiences nursing postoperative children in the CTICU. In-depth individual interviews were conducted in order to get rich information (Korstjens & Moser 2018:121). Data saturation were reached by the tenth participant. To enhance triangulation, the researcher enriched the study’s findings by presenting information gathered from the individual interviews, audio recordings and field notes (Holloway & Galvin 2017:282). The information from the field notes included descriptions of professional nurses’ behaviour, body posture, facial expressions, non-verbal communication, repetition of words, mannerisms and hesitation. These were concurrently analysed with data and used for triangulation (Korstjens & Moser 2018:121).

To ensure transferability, the researcher provided a dense description of the study’s setting, sampling method and direct quotes reflecting professional nurses’ experiences caring for children postoperatively in a CTICU (Korstjens & Moser 2018:121). The researcher also achieved dependability through a dense description of the research methodology and audit trail. Supervisors with extensive qualitative research experience provided guidance during supervision sessions. The study’s dependability was further promoted through meetings with the independent coder, where consensus was reached on the analysed themes and subthemes (Korstjens & Moser 2018:121).

Confirmability is concerned with establishing that the research findings, and interpretation are clearly derived from the collected data, not based on the researcher’s imagination (Korstjens & Moser 2018:121). The researcher had to ensure that NQPNs’ experiences of the research phenomenon were revealed and understood and that there were no interruptions during interviews. The researcher also paid attention to the data that were provided by NQPNs in order to get to the meaning and essence of their experiences (Jang, Yang & Shin 2022:3,4).

## Results

This article describes the following theme: NQPNs’ experiences providing postoperative care to children in a CTICU. The other themes will be retained for subsequent publications.

### Newly qualified professional nurses’ experiences providing postoperative care to children in a cardiothoracic intensive care unit

The following subthemes were linked to this theme: Lack of knowledge and skills to care for children post-cardiac surgery; difficulty understanding treatment modalities and professional nurses experienced fear, anxiety and stress.

#### Lack of knowledge and skills to care for children post-cardiac surgery

The participants cited a lack of knowledge and skills related to their increased workload, insufficient clinical knowledge, lack of adequate communication and difficulties in understanding various cardiac conditions and the surgeries performed on the children in their care. This finding was supported by the following quotes from the participants:

‘I would like to think that my training as a bridging course nurse was sufficient when it comes to adults. However, I do not think that this knowledge applies to children, these children are unique.’ (P1, Age 39 years, Male, Professional Nurse)

Another shocked participant stated:

‘Some of them are still blue, despite surgery being performed. This makes me so frustrated because now I do not understand the logic. To my understanding, they went for surgery, to correct the blue or the cyanosis or the lesion.’ (P7, Age 38 years, Female, Professional Nurse)

Participants shared that professional nurses are expected to act as safe practitioners within their scope of practice to ensure they understand patients’ conditions and treatment. A participant said:

‘You ask the operating doctor why the child is placed on this device. They don’t answer you the way you want, so you continue being unsure of why this child has this type of device. That is the hardest part of nursing a child whose operation you do not understand. ICU-trained professional nurses and doctors should guide, teach, and supervise inexperienced professional nurses to understand different conditions and the surgeries performed on the children in their care. When professionals share perspectives, interpretations of the child’s condition are positively impacted, and there is improved understanding of conditions, leading to professional maturity among the inexperienced nurses.’ (P1, Age 39 years, Male, Professional Nurse)

#### Difficulty understanding treatment modalities

Participants explained that one of the fundamental duties of the professional nurse is administering medication. However, participants further stated that NQPNs lack experience in calculating and administering critical medications such as potassium and inotropes according to CTICU protocol because they do not possess the physiological and pharmacological knowledge required in this setting. They also encounter difficulties in terms of how to communicate their errors.

A participant stated, with a trembling voice:

‘Usually when they start to have the symptoms, sometimes it scares you a bit. Especially if it happens immediately after you have administered medication such as inotropes.’ (P8, Age 42 years, Female, Professional Nurse)

#### Professional nurses experienced fear, anxiety and stress

Participants shared the following challenges during interviews:

‘Nursing children with complex cardiac condition post cardiac surgery can be very difficult and challenging if you are not experienced as we need to support children and their families emotionally. I develop this intense fear and anxiety because I have to manage complications, answerable and accountable to the doctors, management and family for unintentional errors, because I do not have enough information, knowledge and skills to care for these children.’ (P8, Age 42 years, Female, Professional Nurse)

‘You have to do quarterly observations and attend to chest drains make sure that they are patent … I mean the chest drains … monitor haemodynamics and carry out the doctors’ prescriptions … you need to be extra vigilant because the slightest mistake that you commit on the baby, shoo! you can lose your epaulettes.’ (P2, Age 32 years, Female, Professional Nurse)

Newly qualified professional nurses emphasised that they suffered from separation anxiety and temporary attachment, which manifested when they lost either a child or their jobs. The following quotation supports this statement:

‘Working in CTICU is a very stressful because the babies are very sick. … I mean stressful, because post-surgery, the baby will come out with lot of lines, and I don’t understand some of the lines. When we are exposed to increased workloads, we become insecure; this situation adds additional stress on us, and some professional nurses often resign. The shortage of trained and experienced professional nurses often leads to a situation where the unit operates with agency nurses. This intensifies the anxiety and stress experienced in the CTICU, leading to strained interpersonal relationships and communication between nurses and doctors in the CTICU.’ (P3, Age 40 years, Female, Professional Nurse)

A participant shared:

‘But when you are in CTICU you need to know the detailed report of the operation so that you know how to manage the child. Some interventions require you to act immediately regardless of whether the doctor is there or not.’ (P7, Age 38 years, Female, Professional Nurse)

Another participant added:

‘You know when the doctors are around the bedside, they prescribe treatment, but they do not look at me, they are talking to the shift leader, because they trust her, I mean the shift leader. The doctor is looking at her directly, I am a nurse looking after the baby. Maybe they trust her, she knows everything. Sometimes I am thinking because they know her better.’ (P3, Age 40 years, Female, Professional Nurse)

Participants also mentioned that staff should create a trusting relationship and facilitate interdisciplinary communication to ease NQPNs’ fears and anxieties in the CTICU. A participant highlighted that:

‘I do not feel comfortable when there is no shift leader. I feel very lost, I do not know that I feel so empty because I do not have support. I am scared now if anything can happen on this baby.’ (P4, Age 32 years, Female, Professional Nurse)

Some participants supported this statement by asserting that a positive clinical practice environment is critical to meet patients’ health outcomes and the hospital’s strategic objectives. Another participant reflected:

‘I believe trained and experienced professional nurses need to guide and support inexperienced professional nurses in their practice environment to improve their clinical competence. High levels of support and guidance improve professional nurses’ behaviour and afford them a sense of responsibility and accountability. However, negative attitudes and lack of support hinder innovation and increase tension, mistrust, and poor relationships. It is vital that professional nurses in the CTICU support inexperienced nurses in acquiring critical thinking, decision-making, psychomotor and affective skills in the clinical environment; this will ensure the provision of excellent nursing care and assisting each other when nursing critically ill patients.’ (P8, Age 42 years, Female, Professional Nurse)

Most participants agreed that ICU-trained professional nurses should support, supervise and guide inexperienced and newly qualified nurses in their professional development. They should also be involved in clinical teaching to instil confidence in inexperienced professional nurses and promote their ability to interpret policies and protocols appropriately in the clinical setting. ICU-trained professional nurses should use learning opportunities to integrate theory into practice during clinical rounds. However, some ICU-trained professional nurses are reluctant to teach and supervise because of staff shortages and their lack of supervisory and leadership skills.

## Discussion

This article explored and described NQPNs’ experiences providing postoperative care to children in a CTICU and offered recommendations to support them. In the hospital under study in Gauteng province, South Africa, NQPNs are often assigned to work in ICUs because of a severe shortage of trained staff. However, minimal exposure to this environment means they lack sufficient knowledge, encounter challenges in nursing children holistically and cannot make rational decisions and clinical judgements. When NQPNs are equipped with sound clinical judgement, they have the ability to provide care to patients requiring complex interventions postoperatively.

Mentoring is required when working in an ICU and nursing patients on a one-on-one basis. This will allow NQPNs to apply theory and practice under the supervision of an ICU-trained professional nurse. Mentoring and empowerment give them the space to refine their clinical skills, receive guidance and demonstrate their abilities in evaluating patients’ conditions comprehensively following cardiac surgery (Anderson, Moxham & Broadbent 2016:638).

Newly qualified professional nurses should also be exposed to special multidisciplinary units on a rotational basis to empower them with relevant information, problem-solving and decision-making principles, so they maintain a working relationship with various healthcare professionals and are accepted as team members (Falk et al. 2018:2, 6; Qin et al. 2016:451). Newly qualified professional nurses must be able to interpret hemodynamic changes in a patient’s condition to prevent deterioration (Hart et al. 2016:3242).

When NQPNs are sufficiently empowered with knowledge and skills about protocols and procedure manuals on recognising and identifying haemodynamic changes indicating that a child is deteriorating, this will decrease complications, mortality and morbidity rates in critical care settings. In this study, NQPNs stated that they had difficulty interpreting haemodynamic changes in children’s conditions, reflecting their lack of confidence, experience and challenges when intervening, leaving them feeling incompetent and threatened (Esfahani, Varzaneh & Changiz 2016:483).

Providing postoperative care to children in the CTICU requires a thorough understanding of protocols, procedure manuals, surgical interventions and techniques, including children’s anatomy and physiology for best quality care. Bayes and Ewens (2016:599) agreed that future NQPNs need clinical exposure in a CTICU environment to understand the unit’s dynamics. In addition, nursing institutions’ curriculums should cover different paediatric conditions and surgeries to equip nursing students with relevant information, especially in the r. 683 programme. Therefore, there is a need for support services, orientation, induction processes and continuous professional development to prevent the mental and physical stress NQPNs face in this setting (Mikkola, Huhtala & Paavilainen 2016:2961).

Belton (2018:192) reiterated that when NQPNs are assigned to care for children postoperatively, they develop physical, social and emotional side effects like stress, fear and headaches; the unavoidable loss of lives also makes it challenging to interact with patients’ families. Moreover, stress is considered a contributory factor to many physical diseases. Management therefore needs to prioritise and ensure a supportive work environment that enables NQPNs to gain knowledge and holistically improve their skills when caring for postoperative children. Supportive work environments also reduce stress and facilitate high-quality care, which is beneficial to NQPNs and staff (Asadi et al. 2017:75). The NQPNs need to be psychologically and emotionally prepared with good communication skills and form trusting relationships within a team. These attributes encourage positive contributions and promote critical decisions when caring for children postoperatively.

Newly qualified professional nurses’ anxiety can be provoked by professional interactions between nurses and doctors (Karanikola et al. 2016:805). Young, inexperienced professional nurses in the CTICU are prone to anxiety, especially if there is a shortage of ICU-trained professional nurses, increasing their stress (Nooryan et al. 2014:459). However, NQPNs encounter positive and harmonious social relationships with their colleagues when they share their problems, thus decreasing their anxiety levels and promoting professional satisfaction and well-being (Muller & Bester 2016:291).

In addition, when NQPNs work in a complex environment like CTICUs, they become insecure and report that their inability to cope with clinical staff and doctors’ prescriptions often results in their failure to render quality patient care (Mohamedkheir et al. 2016:170, 171). Qin et al. (2016:451) agreed that the ICU environment is stressful because of the responsibility of continuously monitoring patients, which requires nurses to remain vigilant and alert.

Experienced professional nurses should empower NQPNs in CTICUs with relevant skills that allow them to interact with multidisciplinary health team members and acquire more clinical decision-making and problem-solving skills. Support from their colleagues and doctors will enhance their psychological well-being and encourage them to voice their opinions. Enhanced collaboration between professional nurses and doctors also improves lifelong learning opportunities for professional nurses. Slusher et al. (2018:5) affirmed that working together as healthcare professionals in a respectful manner strengthens team cohesion and professional working relationships. Newly qualified professional nurses should learn procedures, protocols and the different standards used in the CTICU environment, while clinical preceptors empower them with sufficient clinical knowledge and skills.

Newly qualified professional nurses should also be allocated enough time and buddied up with ICU-trained professional nurses to increase their ICU competencies (Muthathi, Thurling & Armstrong 2017:6). The buddy system and mentoring would create an opportunity for ICU-trained professional nurses to help NQPNs settle in and adapt to the ICU environment. This support system assists NQPNs to remain focused when they are nursing children postoperatively in CTICUs. This supportive environment allows them to learn at their own pace while developing critical thinking skills and lifelong learning, which enable them to make appropriate decisions and clinical judgements.

### Limitations of the study

The study involved only NQPNs registered under Regulation r. 683 of the SANC in one public academic hospital in Gauteng. Therefore, the findings cannot be generalised. More studies should be conducted with other categories of NQPNs registered under Regulations r. 425, r. 171 and r. 174 of the SANC, as their curriculum includes extensive anatomy, physiology and research components.

### Implications

The study’s findings can be used in planning NQPNs’ orientation and induction into CTICUs. It could also help in developing inclusive teaching strategies in nursing education.

### Recommendations

Recommendations are made based on the study’s findings. These recommendations are specific to nursing practice, nursing research and nursing education.

#### Nursing practice

Newly qualified professional nurses require an orientation programme and in-service training on cardiac abnormalities, conditions in the CTICU and management of children post-cardiac surgery. A rotational ward programme would expose this population to the CTICU environment. A clinical facilitator or shift leader should guide NQPNs regarding the critical aspects of care in the CTICU. Buddying and mentoring systems should also be implemented in the CTICU.

#### Nursing research

Newly qualified professional nurses should be encouraged to read research articles and attend ICU workshops, in-service training and conferences on nursing children post-cardiac surgery for personal and professional development.

#### Nursing education

Additional training, mentoring and follow-up workshops are needed from clinical facilitators and nursing educators to empower NQPNs. Orientation programmes are essential to promote empowerment and professional growth. Simulation and debriefing sessions are also recommended to alleviate NQPNs’ fears and feelings of incompetence; this will assist in clarifying expectations and improving their ICU skills. Nursing education institutions should include a module on nursing children following cardiac surgery.

## Conclusion

The researcher found that NQPNs were experiencing challenges when providing postoperative care to children in a CTICU because of a lack of knowledge and skills, a lack of understanding of different cardiac conditions, surgical interventions and an inability to interpret the child’s condition. This unconducive practice environment creates stress, anxiety and fear. However, a supportive work environment could facilitate NQPNs’ clinical learning, open-mindedness and sound clinical judgement in CTICUs.
